# Magnetic resonance T1-mapping quantitatively assesses the severity of thyroid destruction in patients with autoimmune thyroiditis

**DOI:** 10.3389/fendo.2022.1028588

**Published:** 2022-11-01

**Authors:** Jia Liu, Xiaona Chang, Qiu Wang, Xiaoyu Ding, Tao Jiang, Guang Wang

**Affiliations:** ^1^ Department of Endocrinology, Beijing Chao-Yang Hospital, Capital Medical University, Beijing, China; ^2^ Department of Radiology, Beijing Chao-Yang Hospital, Capital Medical University, Beijing, China

**Keywords:** autoimmune thyroiditis, thyroid T1-mapping, thyroid dysfunction, thyroid destruction, magnetic resonance

## Abstract

**Objective:**

Autoimmune thyroiditis (AIT) is a common organ-specific autoimmune disease. Longitudinal relaxation time mapping (T1-mapping) analyzed by magnetic resonance imaging is a new method for evaluating inflammation or fibrosis. This study aimed to investigate the relationship between thyroid T1-mapping value and degree of intrathyroidal inflammation and destruction in euthyroid AIT patients.

**Methods:**

This case-control study recruited 28 drug-naïve AIT patients and 18 healthy controls. Thyroid specimens were collected for assessing the mRNA expression of inflammatory factors and histopathologic examination. T1-mapping values were measured using a modified look-locker inversion-recovery sequence in all participants.

**Results:**

The positive rate of pathological diagnosed AIT was only 83.3% in the AIT group diagnosed by positive TPOAb and/or TgAb and typical ultrasonic manifestations, while 7.1% of the control group was diagnosed as AIT by pathological manifestations. Receiver operating characteristic curve analysis revealed a very high diagnostic value of thyroid T1-mapping values for pathological diagnosed AIT (area under the curve was 0.950, 95%*CI*: 0.843 – 0.993, *P* < 0.001). In the patients with pathological diagnosed AIT, thyroid T1-mapping values were significantly associated with the mRNA expression of *INF-γ* (*r* = 0.343, *P* < 0.05), *TNF-α* (*r* = 0.352, *P* < 0.01), and *IL-1β* (*r* = 0.673, *P* < 0.01) in thyroid tissues. Moreover, histopathologic examination showed that thyroid T1-mapping values can properly reflect the degree of thyroid destruction in AIT patients.

**Conclusions:**

Thyroid T1-mapping values had a very high diagnostic value for AIT. In euthyroid AIT patients, thyroid T1-mapping values better reflect degree of intrathyroidal inflammation and destruction.

## Introduction

Autoimmune thyroiditis (AIT), a common organ-specific autoimmune disease, is the leading cause of hypothyroidism (1–3). The intrathyroidal pathology of AIT patients is characterized by massive lymphocytes infiltration with germinal center formation, fibrosis, and destruction of the follicular structure (4, 5). AIT patients manifest as different thyroid functional states with varying degree of thyroid destruction (2, 6). Thyroid ultrasound, a major imaging examination for thyroid diseases, often reveals a diffuse hypoechoic or heterogeneous finding in AIT patients (2, 7). However, ultrasound examination cannot quantitatively assess the severity of thyroid destruction in AIT patients (7).

In recent years, magnetic resonance imaging (MRI) is gradually applied to assess thyroid diseases (8–10). Longitudinal relaxation time mapping (T1-mapping) measured by MRI is a new method for evaluating the degree of inflammation or fibrosis in organ or tissues (11–13). Our previous studies showed that overt-hypothyroid AIT patients had increased thyroid T1-mapping values than euthyroid or subclinical-hypothyroid AIT patients (14, 15). And increased thyroid T1-mapping values were corelated with higher TSH and lower FT3 and FT4 levels in AIT patients (14, 15). However, whether thyroid T1-mapping values can evaluate degree of thyroid destruction in AIT patients with normal thyroid function remains unclear. This study aimed to investigate the relationship between thyroid T1-mapping value and degree of intrathyroidal inflammation and destruction in euthyroid AIT patients.

## Materials and methods

### Subjects

A total of 46 patients scheduled for thyroidectomy because of suspicious papillary thyroid carcinoma (less than 1cm in diameter) were recruited from the Otolaryngology Department of Beijing Chao-Yang Hospital, Capital Medical University from April 2021 to January 2022. Patients were included if they met the following criteria (1): meeting the surgical indications (2); normal thyroid function without L-thyroxine substitution (3); receiving neither radiotherapy nor chemotherapy before surgery. The exclusion criteria were as follows (1): claustrophobia or metal implants (2); any chronic inflammatory diseases or other autoimmune disorders (3); use of any hypoglycemic drugs, lipid-lowering agents, immunosuppressive drugs, or steroid treatment (4); acute or chronic infectious disease (5); pregnancy, possibly pregnancy or ingesting agents known to influence thyroid function (6); other co-existing malignant disease (7); coronary disease, heart failure, chronic obstructive pulmonary disease, liver or kidney disease. The clinical characteristics including free T3 (FT3), free T4 (FT4), thyroid-stimulating hormone (TSH), antithyroid peroxidase antibody (TPOAb), antithyroglobulin antibody (TgAb), and thyroid ultrasound were collected in all participants. Normal thyroid function was defined when thyroid function, including FT3, FT4, and TSH, were in the normal ranges (normal range: FT3: 2.63–5.71 pmol/L; FT4: 9.10–19.2 pmol/L; TSH: 0.35–4.94 mIU/L). The detection range for TPOAb and TgAb) was 28-6500 IU/mL and 15-2500 IU/mL, respectively. Positive for TPOAb or TgAb was diagnosed as the value > 60 IU/mL (normal range for TPOAb or TgAb: 0.00~60.0 IU/mL). AIT was diagnosed by positive for TPOAb and/or TgAb and typical hypoechoic or heterogeneous finding in a thyroid ultrasound (2). The healthy control subjects were defined based on the following criteria: normal thyroid function; both TPOAb and TgAb were negative; and thyroid ultrasound was normal.

The present study complied with the Helsinki Declaration, and the protocol was approved by the Ethics Committee of the Beijing Chao-Yang Hospital, Capital Medical University. All enrolled subjects provided written informed consent.

### Clinical and biochemical measurements

The same trained group measured height and weight, and collected information about health status and medications of all participants. Height and weight were measured to the nearest 0.1 cm and 0.1 kg, respectively. BMI was calculated as weight in kilograms divided by height in meters squared. Venous blood samples were obtained from all the participants after overnight fasting and before surgery. The parameters regarding thyroid function (FT3, FT4 and TSH), thyroid antibodies (TPOAb and TgAb), and hsCRP were analyzed immediately. The serum was separated by centrifugation at 3000 r/min for 10 min at 4°C and was stored at −80°C for serum inflammatory cytokines assays. FT3, FT4 and TSH were measured by electrochemiluminescence immunoassay using an Abbott Architect i2000 (Dimension Vista, Siemens Healthcare Diagnostics, Germany). The serum concentrations of TPOAb and TgAb were detected by chemiluminescent immunoassay (Dimension Vista, Siemens Healthcare Diagnostics, Germany). High sensitivity C reactive protein (hsCRP) was measured by immunonephelometric assay. A well-trained ultrasound physician assessed the thyroid ultrasound.

### Thyroid specimens

Thyroid specimens were collected from at least 2cm away from the nodule. A part of thyroid tissue specimen was stored at −80°C within 30 minutes for total RNA extraction. And the other part of thyroid tissue specimen was fixed in 10% formalin for 24 hours to make paraffin-embedded blocks further, cut into 3 μm thick serial sections, and finally subjected to Hematoxylin and Eosin staining (H&E) using a standard protocol.

The pathological diagnosis of AIT was based on the HE characteristics with massive lymphocytes infiltration with germinal center formation, and destruction of the follicular structure.

### Cell RNA extraction and qRT-PCR

Total RNA was extracted from thyroid tissues by TRIzol (T9424, Sigma, USA) and phenol-chloroform. The concentration and purity of the extracted total RNA were subsequently determined using a Nanodrop 2000C spectrophotometer (Nano- Drop Technologies, USA), and samples with A260/A280 ranging between 1.8 and 2.0 were used in the experiments. Reverse transcription reactions were carried out by HiScript III All-in-one RT SuperMix Kit (R333, Vazyme, China). RT-qPCR amplifications were performed on 7500 Real-Time PCR System (Applied Biosystems, USA) using a SYBR Green Kit (Q711, Vazyme, China) according to the manufacturer´s instructions, and β-actin gene was used as an internal reference (forward, 5´-GCCGCCAGCTCACCAT-3´ and reverse, 5´-TCGTCGCCCACATAGGAATC-3´). The specific primers are as follows: *INF-γ* forward, 5´-AGTGATGGCTGAACTGTCGC-3´ and reverse, 5´-ACTGGGATGCTCTTCGACCT-3´; *TNF-α* forward, 5´-TCTCCTTCCTGATCGTGGCA-3´ and reverse, 5´-CAGCTTGAGGGTTTGCTACAAC-3´; *IL1β* forward, 5´-CCAGGGACAGGATATGGAGCA-3´ and reverse, 5´-TTCAACACGCAGGACAGGTACAG-3´.

### Enzyme-linked immunosorbent assay

According to the manufacturer´s instructions, the concentration of *IL-1β* in human serum was detected by ELISA using commercial quantitative kits (Abcam, ab214025, UK). All samples were measured using a microplate reader (Thermo Fisher Scientific, USA) capable of measuring absorbance at 450 or 600 nm. By plotting the relationship between the absorbance values and the gradient concentration of the standard product provided by the kit, the standard curves were generated during the determination process.

### Thyroid magnetic resonance imaging

Just as our previous studies described, all participants had a thyroid MRI in a supine position using 3 Tesla scanner on a Tim Trio system (Siemens Healthcare, Erlangen, Germany) within one week before having a thyroidectomy (14, 15). Images were obtained by gap-free full thyroid coverage with a slice thickness of 8mm. All routine imaging and maps were analyzed using the Argus (SYNGO MMWP Workstation, Siemens AG). Two experienced radiologists blinded to the groups performed image analyses for thyroid T1-mapping value using a modified look-locker inversion-recovery (MOLLI) sequence (16). T1 mapping values for each thyroid lobe were calculated from the average of all sections on that side.

### Statistical analysis

Data were analyzed using SPSS 22.0 (SPSS, Chicago, IL, USA) and MedCalc 15.10 (MedCalc Software, Mariakerke, Belgium). The distribution of continuous data was analyzed using the Kolmogorov‐Smirnov test. Normally distributed data were expressed as mean ± standard deviation, while variables with a skewed distribution including TSH, TPOAb, TgAb, hsCRP and *IL-1β* were given as the median and upper and lower quartiles. The differences between two groups were analyzed using an independent sample t-test or a Mann-Whitney U test. The proportions were analyzed using chi-squared tests. Correlation analyses were performed using Pearson and Spearman correlations. A receiver operating characteristic (ROC) curve was used to evaluate the diagnostic performance of thyroid T1-mapping value for the degree of thyroid destruction, and Youden index was used as a criterion for selecting the optimum cutoff point using MedCalc 15.10. Statistical significance was considered with two-tailed analyses as *P* < 0.05.

## Results

### Baseline characteristics of the control and AIT groups

The baseline characteristics of the control and AIT groups were showed in **Table 1**. There was no significant difference in age, gender, and BMI levels between the two groups. The levels of FT3, FT4 and TSH were also statistically the same between the control and AIT groups. No significant difference in the circulating inflammatory factors, including hsCRP and *IL-1β*, was observed between the control and AIT groups. The AIT patients had increased levels of TPOAb and TgAb than the controls (*P* < 0.05; **Table 1**). Thyroid T1-mapping values of the AIT patients were significantly increased when compared with the control group (1030.4 ± 80.7 *vs.* 911.2 ± 85.1 ms; *P* < 0.01; **Table 1**). Interestingly, the positive rate of pathological diagnosed AIT was 7.1% in the control group and 83.3% in the AIT group (*P* < 0.01; **Table 1**).

**Table 1:** The baseline characteristics of the control and AIT groups.

Parameters	Control group (n = 28)	AIT group (n = 18)	*P*
**Age,y**	40.7 ± 9.3	44.1 ± 14.2	.376
**Gender, Males/Females, n**	5/23	4/14	.721
**BMI, kg/m^2^**	23.69 ± 3.81	24.37 ± 3.24	.528
**FT3, pmol/L**	5.01 ± 0.40	4.99 ± 0.39	.834
**FT4, pmol/L**	15.31 ± 1.65	15.21 ± 1.68	.833
**TSH, μIU/mL**	1.74 (1.17 – 2.36)	1.83 (1.33 – 2.89)	.196
**TPOAb, IU/mL**	28.00 (28.00 – 31.82)	52.05 (28.00– 1850.58)	.016
**TgAb, IU/mL**	15.20 (15.00 – 21.00)	140.80 (61.50 – 222.88)	.000
**hsCRP, mg/L**	0.59 (0.29 – 1.23)	0.57 (0.23 – 0.88)	.507
**IL-1β, pg/mL**	31.18 (28.31 – 37.12)	32.33 (28.11 – 35.21)	.988
**T1-mapping value, ms**	911.2 ± 85.1	1030.4 ± 80.7	.000
**Pathological diagnosis positivity, n (%)**	2 (7.1)	15 (83.3)	.000

Data are means ± SD unless indicated otherwise. TSH, TPOAb, TgAb, hsCRP and IL-1β are shown as median, upper, and lower quartiles. BMI: body mass index; FT3, free T3; FT4, free T4; TPOAb, antithyroid peroxidase antibodies; TgAb, antithyroglobulin antibodies; hsCRP, high sensitivity C reactive protein; IL-1β, interleukin-1β; AIT, autoimmune thyroiditis.

### Correlation between thyroid T1-mapping values and clinical parameters

Thyroid T1-mapping values were significantly associated with the serum thyroid antibodies, including TPOAb and TgAb, in all participants (TPOAb: *r* = 0.412, *P* < 0.01; TgAb: *r* =0.579, *P* < 0.01; **Table 2**). However, there was no significant correlation between thyroid T1-mapping values and other clinical parameters, including age, BMI, FT3, FT4, TSH, hsCRP and *IL-1β* (**Table 2**).

**Table 2:** Correlation between thyroid T1-mapping values and clinical parameters.

Parameters	T1-thyroid (All)		T1-thyroid (Control)		T1-thyroid (AIT)	
	*r*	*P*	*r*	*P*	*r*	*P*
**Age**	.209	.162	.158	.442	.449	.062
**BMI**	.066	.664	-.049	.804	.135	.595
**FT3**	-.024	.876	-.010	.961	.111	.660
**FT4**	-.054	.723	-.140	.476	.234	.349
**TSH**	-.106	.485	-.209	.287	-.365	.137
**TPOAb**	.412	.004	.284	.143	.166	.510
**TgAb**	.579	.000	.170	.386	.368	.133
**hsCRP**	-.117	.449	-.218	.275	.010	.996
**IL-1β**	.075	.622	-.250	.200	.538	.021

BMI, body mass index; FT3, free T3; FT4, free T4; TPOAb, antithyroid peroxidase antibodies; TgAb, antithyroglobulin antibodies; hsCRP, high sensitivity C reactive protein; IL-1β, interleukin-1β; AIT, autoimmune thyroiditis.

However, there was no significant association between thyroid T1-mapping values and serum thyroid antibodies in the control or AIT groups, respectively. In the AIT group, the positive association between thyroid T1-mapping values and serum *IL-1β* levels was observed (*r* = 0.538, *P* < 0.05; **Table 2**).

### Diagnostic performance of thyroid T1-mapping values for AIT

**Figure 1:**
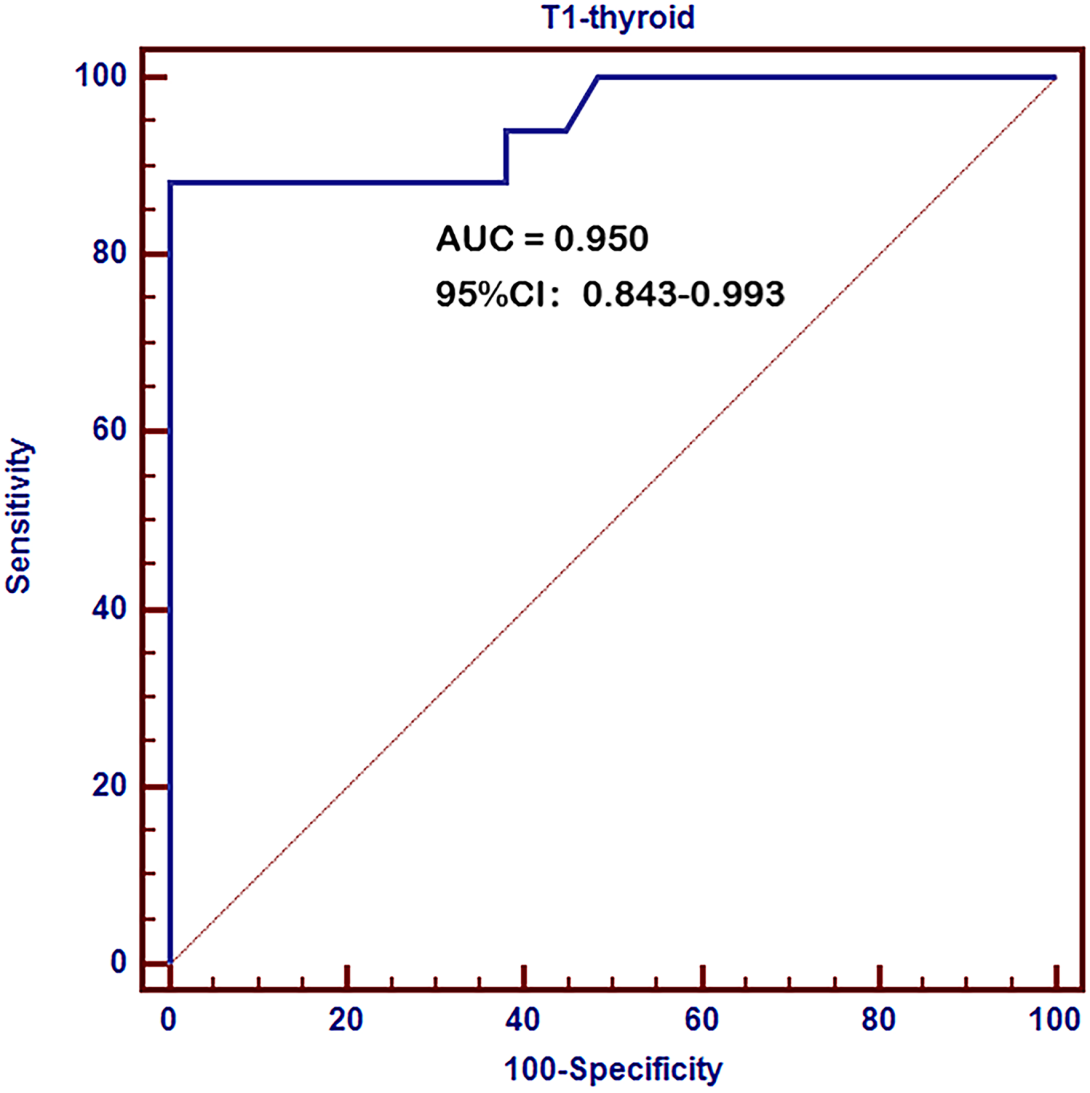
Receiver operating characteristic (ROC) curves of thyroid T1-mapping values for pathological diagnosed AIT. The area under the curve (AUC) was 0.950 (95%CI: 0.843 – 0.993, *P* < 0.001). The optimal cutoff point estimated from Youden index was 997, with a sensitivity of 88.2% and a specificity of 100.0%.

ROC curve analysis revealed a very high diagnostic value of thyroid T1-mapping values for pathological diagnosed AIT. The area under the curve (AUC) was 0.950 (95%CI: 0.843 – 0.993, *P* < 0.001, **Figure 1**). The optimal cutoff point estimated from Youden index was 997, with a sensitivity of 88.2% and a specificity of 100.0%.

### Correlation between thyroid T1-mapping values and the expression of inflammatory factors in thyroid tissues

**Figure 2:**
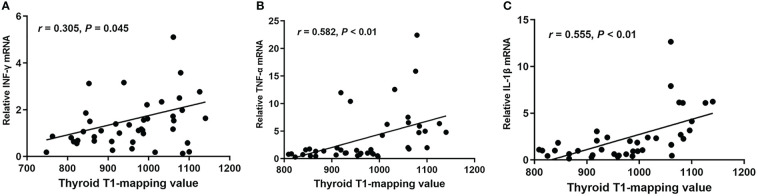
The correlation between thyroid T1-mapping values and the mRNA expression of *INF-γ* **(A)**, *TNF-α* **(B)**, and *IL-1β* **(C)** in thyroid tissues. Thyroid T1-mapping values were significantly associated with the mRNA expression of *INF-γ*, *TNF-α*, and *IL-1β* in thyroid tissues (*INF-γ*: *r* = 0.305, *P* = 0.045; *TNF-α*: *r* = 0.582, *P* < 0.01; *IL-1β*: *r* = 0.555, *P* < 0.01). A bivariate correlation analysis was performed using the Spearman test. *r* represents the Spearman correlation coefficient.

In order to investigate whether thyroid T1-mapping values were related to degree of intrathyroidal inflammation, we measured the mRNA expression of inflammatory factors in thyroid tissues, including *INF-γ*, *TNF-α*, and *IL-1β*. Thyroid T1-mapping values were significantly associated with the mRNA expression of *INF-γ*, *TNF-α*, and *IL-1β* in all participants (*INF-γ*: *r* = 0.305, *P* = 0.045; *TNF-α*: *r* = 0.582, *P* < 0.01; *IL-1β*: *r* = 0.555, *P* < 0.01; **Figure 2**). We further evaluated the association between thyroid T1-mapping values and the mRNA expression of inflammatory factors in thyroid tissues in the patients with pathological diagnosed AIT. Thyroid T1-mapping values were significantly associated with the mRNA expression of *INF-γ*, *TNF-α*, and *IL-1β* (*INF-γ*: *r* = 0.343, *P* < 0.05; *TNF-α*: *r* = 0.352, *P* < 0.01; *IL-1β*: *r* = 0.673, *P* < 0.01). However, there was no significant association between thyroid antibodies (TPOAb and TgAb) and the mRNA expression of inflammatory factors in thyroid tissues in the patients with pathological diagnosed AIT (*P* > 0.05).

### Correlation between thyroid T1-mapping values and thyroid destruction measured by histopathologic examination

**Figure 3:**
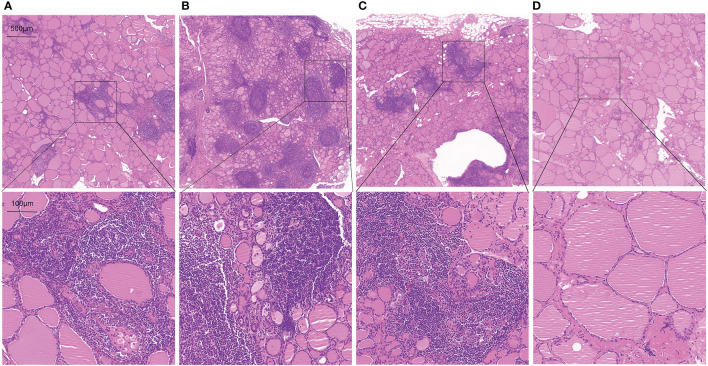
Representative HE staining images in thyroid tissue sections from AIT patients (up:20× magnification, down:100× magnification). **(A)** female, 49 years old, TPOAb < 28 U/ml, TGAb = 199.9 U/ml, T1 = 979. **(B)** female, 30 years old, TPOAb = 1934.5 U/ml, TGAb = 179.9 U/ml, T1 = 1060. **(C)** female, 49 years old, TPOAb < 28 U/ml, TGAb = 75 U/ml, T1 = 1006. **(D)** male, 52 years old, TPOAb = 33.8 U/ml, TGAb = 291.8 U/ml, T1 = 766.

To further assess whether thyroid T1-mapping values reflected the degree of thyroid destruction, we took HE staining images in thyroid tissue sections from the AIT patients. As shown in **Figure 3**, thyroid T1-mapping values can properly reflect the degree of thyroid destruction in AIT patients. Although with similar slightly elevated thyroid antibodies (**Figures 3A** **, C, D**), the three patients had different thyroid T1-mapping value and the degree of thyroid destruction.

## Discussion

The present study showed that the positive rate of pathological diagnosed AIT was only 83.3% in the AIT group diagnosed by positive TPOAb and/or TgAb and typical ultrasonic manifestations, while 7.1% of the control group was diagnosed as AIT by pathological manifestations. ROC curve analysis revealed a very high diagnostic value of thyroid T1-mapping values for pathological diagnosed AIT. In the patients with pathological diagnosed AIT, thyroid T1-mapping values were significantly associated with the mRNA expression of *INF-γ*, *TNF-α*, and *IL-1β* in thyroid tissues. Moreover, histopathologic examination showed that thyroid T1-mapping values can properly reflect the degree of thyroid destruction in AIT patients.

According to different degree of thyroid destruction, AIT patients manifest as different thyroid functional states, and most of them may undergo a long term of normal thyroid function (2, 6). When AIT patients had hypothyroidism, thyroid function can be used to indirectly reflect the degree of thyroid destruction. However, so far there is still lack of an imaging technique for assessing the degree of thyroid destruction in AIT patients with normal thyroid function. Usually, AIT was diagnosed by positive thyroid antibodies (TPOAb and/or TgAb) and typical hypoechoic or heterogeneous manifestations of thyroid ultrasound (2, 17, 18). The present study showed that the positive rate of pathological diagnosed AIT was only 83.3% in the AIT group diagnosed by positive TPOAb and/or TgAb and typical ultrasonic manifestations, while 7.1% of the control group was diagnosed as AIT by pathological manifestations. Therefore, as a major imaging technique for thyroid diseases, thyroid ultrasound seems to lack sensitivity and specificity. Furthermore, thyroid ultrasound cannot quantitatively evaluate the degree of thyroid destruction (7). There is an urgent need for a new technique for assessing intrathyroidal destruction in AIT patients, especially euthyroid AIT patients.

T1-mapping measured by MRI is a new technique for assess the degree of inflammation or fibrosis (11–13). Our previous studies showed that overt-hypothyroid AIT patients had increased thyroid T1-mapping values than euthyroid or subclinical-hypothyroid AIT patients (14, 15). And increased thyroid T1-mapping values were corelated with higher TSH and lower FT3 and FT4 levels in AIT patients (14, 15). In the present study, ROC curve analysis revealed a very high diagnostic value of thyroid T1-mapping values for pathological diagnosed AIT. Therefore, thyroid T1-mapping values might be a better technique for identifying AIT.

Immune abnormality mediated by lymphocytes, especially T lymphocytes, play an important role in the pathogenesis of AIT (5, 19). Infiltration of T lymphocytes formed germinal center, promoted the production of inflammatory factors, such as *INF-γ*, *TNF-α*, and *IL-1β*, and further caused fibrosis and destruction of follicular structure in AIT patients (5, 19). In the patients with pathological diagnosed AIT, thyroid T1-mapping values were significantly associated with the mRNA expression of *INF-γ*, *TNF-α*, and *IL-1β* in thyroid tissues. Both TPOAb and TgAb, mainly produced by lymphocytes infiltrated in thyroid, are commonly used as diagnostic markers for AIT in clinical practice (2, 5, 20). In the present study, the AIT patients had increased levels of TPOAb and TgAb than the controls. However, there was no significant association between thyroid antibodies (TPOAb and TgAb) and the mRNA expression of inflammatory factors in thyroid tissues in the patients with pathological diagnosed AIT. In the present study, although with similar slightly elevated thyroid antibodies (**Figures 3A, C, D**), the three patients had different thyroid T1-mapping value and the degree of thyroid destruction. Therefore, these results might suggest that thyroid T1-mapping values better reflect degree of intrathyroidal inflammation and destruction in euthyroid AIT patients.

Several limitations of this study should be mentioned. First, this study was a case-control study with a relatively small sample size. The generality of these results from the present study was restricted. Further studies with a large sample size were needed to confirm the results. Second, because it is pretty hard to get thyroid tissue from AIT patients and healthy controls, we collected thyroid specimens from at least 2cm away from papillary thyroid microcarcinoma. Therefore, it cannot entirely exclude the influence of tumor. Moreover, MRI is a relatively expensive detection method, so the cost-benefit ratio should be considered. Nevertheless, the present study provides a new perspective and direction for quantitatively assessing degree of intrathyroidal inflammation and destruction in euthyroid AIT patients. AIT patients with higher thyroid T1-mapping values may be likely to developing hypothyroidism, and should be paid more attention and take careful follow-up examination.

In conclusions, thyroid T1-mapping values had a very high diagnostic value for AIT. In euthyroid AIT patients, thyroid T1-mapping values better reflect degree of intrathyroidal inflammation and destruction.

## Data availability statement

The raw data supporting the conclusions of this article will be made available by the authors, without undue reservation.

## Ethics statement

The studies involving human participants were reviewed and approved by Ethics Committee of the Beijing Chao-Yang Hospital, Capital Medical University. The patients/participants provided their written informed consent to participate in this study.

## Author contributions

JL, TJ and GW conceptualized and designed the study. XC and QW performed T1-mapping and thyroid destruction experiments and acquired data. XD performed analysis and interpretation of the data. JL wrote the manuscript. All authors contributed to the article and approved the submitted version.

## Funding

This work was supported by grants from the Beijing Hospitals Authority Clinical Medicine Development of Special Funding Support (grant number ZYLX202106).

## Acknowledgments

We thank all study participants for their cooperation.

## Conflict of interest

The authors declare that the research was conducted in the absence of any commercial or financial relationships that could be construed as a potential conflict of interest.

## Publisher’s note

All claims expressed in this article are solely those of the authors and do not necessarily represent those of their affiliated organizations, or those of the publisher, the editors and the reviewers. Any product that may be evaluated in this article, or claim that may be made by its manufacturer, is not guaranteed or endorsed by the publisher.
